# Drivers and Pathways for the Recovery of Critical Metals from Waste‐Printed Circuit Boards

**DOI:** 10.1002/advs.202309635

**Published:** 2024-06-05

**Authors:** Dong Xia, Carmen Lee, Nicolas M. Charpentier, Yuemin Deng, Qingyu Yan, Jean‐Christophe P. Gabriel

**Affiliations:** ^1^ SCARCE Laboratory Energy Research Institute @ NTU Nanyang Technological University Singapore 639798 Singapore; ^2^ School of Material Science and Engineering Nanyang Technological University Singapore 639798 Singapore; ^3^ Université Paris‐Saclay CEA CNRS NIMBE LICSEN Gif‐sur‐Yvette 91191 France; ^4^ Ecologic France 15 Avenue du Centre Guyancour 78280 France

**Keywords:** artificial intelligence, catalysts, critical metals, green metallurgy, hyperspectral sensing, waste printed circuit board

## Abstract

The ever‐increasing importance of critical metals (CMs) in modern society underscores their resource security and circularity. Waste‐printed circuit boards (WPCBs) are particularly attractive reservoirs of CMs due to their gamut CM embedding and ubiquitous presence. However, the recovery of most CMs is out of reach from current metal‐centric recycling industries, resulting in a flood loss of refined CMs. Here, 41 types of such spent CMs are identified. To deliver a higher level of CM sustainability, this work provides an insightful overview of paradigm‐shifting pathways for CM recovery from WPCBs that have been developed in recent years. As a crucial starting entropy‐decreasing step, various strategies of metal enrichment are compared, and the deployment of artificial intelligence (AI) and hyperspectral sensing is highlighted. Then, tailored metal recycling schemes are presented for the platinum group, rare earth, and refractory metals, with emphasis on greener metallurgical methods contributing to transforming CMs into marketable products. In addition, due to the vital nexus of CMs between the environment and energy sectors, the upcycling of CMs into electro‐/photo‐chemical catalysts for green fuel synthesis is proposed to extend the recycling chain. Finally, the challenges and outlook on this all‐round upgrading of WPCB recycling are outlined.

## Graphical abstract

This review highlights the critical role of waste‐printed circuit boards in preserving valuable critical metals (CMs) for resource security. By identifying 41 underutilized CMs, the review explores innovative pathways for their recovery, including advanced enrichment strategies and greener recycling methods. The study also proposes upcycling CMs into catalysts for green fuel synthesis to extend the recycling chain.

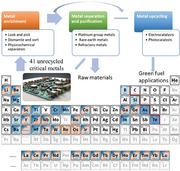

## 1 Introduction

Because of utilizing virtually every stable element in the periodic table for its unique physical and chemical properties, many modern electronic products are now more functional and reliable than before.^[^ ^1^ ^]^ Among these elements, critical metals (CMs), also known as strategic metals or critical minerals, are a group of elements that are indispensable to a country's economic development, closely related to geopolitics, and essential to the industrial base and modern technologies such as information technology, aerospace, and renewable energy. Due to their limited availability and potential risk of supply chain disruptions, many countries and regions have highlighted their important position and security significance since the new century.^[^ ^2^ ^]^ In addition, the outbreak of the COVID‐19 pandemic and regional conflicts in recent years restricted international traffic and material flow, causing some mines to temporarily close thus exacerbating supply and demand problems. As a result, the importance of finding alternative sources for CMs is becoming increasingly urgent.

Urban mining of CMs has gained surging attention as it can increase the security and circularity of strategic resources and reduce the negative geopolitical/environmental impacts and energy consumption associated with primary mining.^[^ ^3^ ^]^ E‐waste has been recognized as a high‐priority waste stream worldwide under the United Nations’ guidance since 2002 and its management has been profoundly influenced by the entry into force of the Basel Convention.^[^ ^4^ ^]^ In 2022, the global quantity of e‐waste generated was ≈62 million tonnes and is projected to increase to 82 million tonnes by 2030.^[^ ^5^ ^]^ Accordingly, the e‐waste metal recovery market is projected to grow from USD 59 billion to USD 140 billion during the period, at a Compound Annual Growth Rate (CAGR) of 12.9%.^[^ ^6^ ^]^ To e‐waste recyclers, waste ‐ printed circuit boards (WPCBs) are the most attractive secondary resource, due to their universal presence in nearly all electronic products and the valuable metals they contain, such as gold, silver, copper, and platinum group metals (PGM), which constitute more than 80% of the total recycling revenue, despite only representing 4–7% of their weight.^[^ ^7^ ^]^ However, the increasing complexity and diversity of modern PCB manufacturing have created a conundrum for recycling: the more intricate the product, the more challenging to reclaim and preserve the highly processed resources used to create it.^[^ ^8^ ^]^ Furthermore, the use of diverse CMs is increasing overwhelmingly as required by electronic advancements. Given the quantities of electronic devices and their generally short lifetimes, end‐of‐life losses of CMs will also increase sharply unless advances in recycling/upcycling keep pace with the development of sophisticated electronic technology.

Numerous efforts have been made to advance the sustainability of PCBs within the context of a circular economy for the last three decades, leading mainstreamed recycling processes from scattered informal workshops to large‐scale industries.^[^ ^9^ ^]^ As the major economic driver for PCB recycling is from the recovery of a limited species of metallic materials, the state‐of‐art PCB recycling is mostly metal‐centric, integrating physical separation, pyrometallurgy, hydrometallurgy, and bio‐metallurgy to obtain metal concentrates and refined metallic products.^[^ ^10^ ^]^ Currently, industrial mass recycling still favors pyrometallurgy, e.g., smelting, to recycle some precious and base metals, with hydrometallurgical and/or electrochemical processes deployed subsequently for further metal separation and refining.^[^ ^11^ ^]^ The hydrometallurgical options start to play a more important role due to low capital costs, precise control, high chemical efficiency, and flexibility to various elements.^[^ ^12^ ^]^ Many signs of progress have been made as well on a laboratory or pilot scale to promote efficiency and environmental friendliness, such as in supercritical fluid extraction,^[^ ^13^ ^]^ and ionic liquid or deep eutectic solvent processing.^[^ ^14^ ^]^ In addition, to extend the e‐waste recycling chain and further increase the recycling revenue, some methods were developed to upcycle materials from waste to value‐added products through regulating (micro or macro) structures and endow properties for catalytic,^[^ ^15^ ^]^ optical,^[^ ^16^ ^]^ and other applications.^[^ ^17^ ^]^

Even so, of the 70 elements that make up PCBs, only a dozen metals are commonly recycled by industries, whereas a large number of remaining elements categorized in the group of CMs have rarely received the attention they deserve owing to techno‐economic feasibility constraints.^[^ ^18^ ^]^ In recent years, with the rapid development of hyperspectral sensing, artificial intelligence （AI） recognition, and green metallurgical technology, the PCB recycling industry has ushered in all‐round upgrade opportunities. Meanwhile, a paradigm‐shifting recycling route, including automatic dismantling, advanced sorting, physicochemical separation, and metallurgical purification is being prompted to identify more CMs with recycling potential since it can provide a continuous entropy reduction for all elements embedded in WPCBs throughout the recycling route.^[^ ^19^ ^]^ Furthermore, in light of CMs being the vital substantial nexus between the environment and energy sectors, a huge demand for CM‐based energy catalysts provides a supplementary pathway to convert CMs from grave to cradle with higher cost‐benefit efficiency. All these pathways should contribute to delivering a full‐palette CM recovery from WPCBs to reach the ultimate zero‐waste scheme. Therefore, a visionary review is in demand on the effective integration of these advanced technologies into the new PCB recycling pathways.

This review begins with a defined scope of CMs from a global and historical perspective to prioritize CMs for the paradigm‐shifting recovery process. Then three main strategies for metal enrichment, “dismantle and sort”, “look and pick”, and physicochemical separation are compared regarding practicability and effectiveness, focusing on current development and proper deployment of AI algorithms, hyperspectral sensing, X‐ray‐transmission‐K‐edge detection, and ultrafast electrical heating to optimize the initial entropy reduction steps. This is followed by a comprehensive discussion of tailored metallurgical methods for the recovery of precious, rare earth, and refractory metals to tradable products, with an emphasis on highly selective and sustainable methods based on individual intrinsic physicochemical discrepancies. Furthermore, an upcycling strategy of CMs from WPCBs into electro‐/photo‐chemical catalysts for green fuel synthesis is proposed, and associated catalytic fundamentals, synthetic methods, and catalytic applications are discussed. Finally, the outlooks on this all‐round upgrading of PCB recycling are provided toward a higher level of CM sustainability.

## 2 Scope of Critical Metals

### 2.1 Critical Metals from a Global Scale

**Figure 1:**
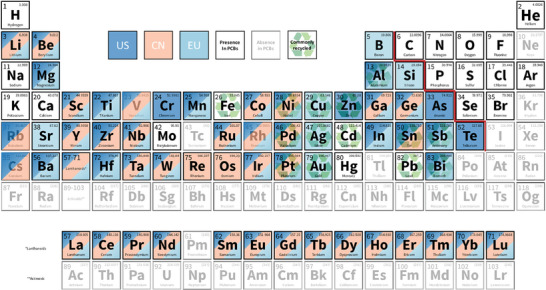
Periodic table showing the common CMs designated by three governing bodies (the US, China, and EU), elements present in PCBs, and metals that have been commonly recycled.

First, it is essential to have a well‐defined scope of CMs from a contemporary global scale considering its geographical diversity and temporal evolution. CMs refer to metallic elements (sometimes including metalloids) among critical minerals. They have two remarkable characteristics: essential for the functioning of modern technologies, economies, or national security, and vulnerable to potential disruptions of supply chains.^[^ ^20^ ^]^ In recent years, considering their national/regional development strategy, many countries and regions updated critical material lists in succession, including Australia, Canada, China, South Korea, the United Kingdom, the United States, and the European Union. A compilation of some representative lists is illustrated within the periodic table of elements in **Figure** **1**.^[^ ^21^ ^]^

Entering the 21st century, the US government started issuing a critical material strategy, where materials’ contribution to clean energy and supply risk became two evaluation indicators for criticality. The most up‐to‐date list was released by the United States Geological Survey in 2022, including 50 critical mineral commodities.^[^ ^22^ ^]^ Furthermore, the list will be updated at least every three years to dynamically represent current supply, demand, concentration of production, and policy priorities.

Within Europe, reliable and unhindered access to certain raw materials is also a growing concern. To address this challenge, the European Commission has created a list of critical raw materials for the EU every three years since 2011. The newest version (fifth) was published in 2023 in the Study on the Critical Raw Materials for the EU 2023‐Final Report.^[^ ^23^ ^]^ The list includes 34 critical raw minerals, reflecting current production, market, and technological developments. The UK's first‐ever critical minerals strategy promulgated in 2022 included 18 minerals with high criticality and 5 minerals on a “watchlist” to improve the security of the supply of critical minerals for the UK.^[^ ^24^ ^]^ The cohort of minerals is largely covered in the EU list except for tellurium and tin.

Both the Canadian and Australian Governments have established the lists as well with the designation of 26 and 31 resource commodities as critical minerals, respectively.^[^ ^25^ ^]^ As major resource suppliers, they pay great attention to their geological endowment and the sustainability of resource supply to allies and partners in addition to economic importance, supply risks, and low‐carbon transition requirements. Therefore, their lists of critical minerals are largely covered in those found in the US and the EU catalogs.

As countries heavily import‐dependent on natural resources, Japan and South Korea have long approached the security of critical minerals and materials supply chains as a top priority. Japan has updated since 2014 to include now 34 critical minerals and two mineral groups—PGMs and rare earth elements (REEs).^[^ ^26^ ^]^ South Korea announced a list of 33 critical minerals concerning economic security.^[^ ^27^ ^]^ All CMs in both lists are covered in Figure 1 except for uranium.

The structure of China's industry is notably different from other developed regions, which makes it difficult to directly apply the criticality criteria that have been used in Europe and the US. The only official list up to now comprising 21 strategic minerals for China was issued in the “National Mineral Resources Plan (2016–2020)” in 2016.^[^ ^28^ ^]^ To address the latest criticality assessment, some scientific groups have made many efforts to identify CMs, among which a 3D methodology was developed considering supply safety, domestic economy, as well as environmental risk, and it developed 37 metals with a high criticality degree among 64 materials.^[^ ^29^ ^]^

### 2.2 Elemental Constitution in PCBs

Just as the list of CMs is evolving with time, so is the elemental constitution in PCBs. However, the production dates of PCBs in their end‐of‐life stockpile can often date back years or even decades, depending on their ages and disposal methods. Given that recycling activities often lag production on the timeline, a good understanding of CM reserves deposited in PCBs from a historical perspective can help stakeholders make better decisions to mitigate criticality issues and take recycling measures in advance.

PCBs can be found in nearly all electronic products as an integrated and functional system to connect various electronic components (ECs) in a controlled manner.^[^ ^30^ ^]^ A typical PCB has a laminated structure of conductive and insulating layers. ECs refer to any fundamental, separate device or physical entities that influence electrons or their corresponding fields. ECs are fixed to conductive pads on the outer layers, which are designed to accept the ECs' terminals, and then soldered to both electrically connect and mechanically fasten them to the board. These ECs can be categorized as passive, active, or electromechanical. After a bare PCB is populated with various ECs to achieve its performance and functionality, it is often called a printed circuit assembly, or printed circuit board assembly, and the terms “PCB” or “PCBA” usually refer to them.

PCBs have undergone significant changes in their elemental constitution over the years, driven by advances in technology and changing environmental regulations. A prime example given by Johnson et al.^[^ ^31^ ^]^ showed this evolution of the elemental constitution in Inter PCBs. Earlier PCBs only contained a few types of metals, such as Cu and Ni, due to a simple design. Starting in the 1980s, there was a trend towards using smaller surface‐mounted parts in place of through‐hole ones, resulting in PCBs with smaller footprints and reduced production costs. In the 1990s, the use of multilayer PCBs became more common, allowing for the integration of more intricate circuits into even smaller areas. As a result of this advancement, there was increased utilization of metals such as Au, Ag, and Sn in the manufacturing procedure. It also came with concerns about the environmental pollution of hazardous materials used in PCBs, which led to the development of new materials and manufacturing techniques that reduced or eliminated the use of Pb, Hg, and other toxic substances. By the 2000s, the number of elementals in PCBs had ballooned to up to 70, which is basically the level of modern PCBs.^[^ ^10^, ^32^ ^]^ In the last two decades, more and more CMs have played a vital role in PCBs with the proliferation of revolutionary technologies such as advanced communications, AI, the internet of things, wearable facilities, big data, and cloud computing, to name a few.^[^ ^33^ ^]^ All possible elements present in modern PCBs are also summarized in the periodic table (Figure 1).

**Figure 2:**
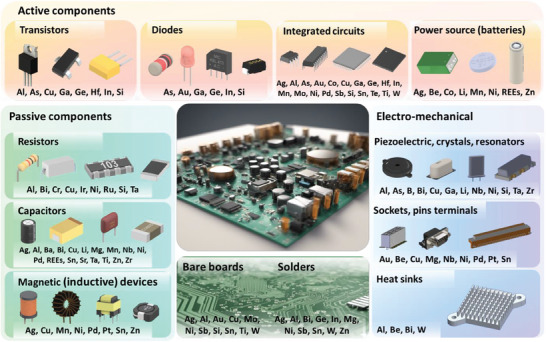
Component composition and the corresponding CM constitution in a typical PCB. Non‐CMs, Pb and Fe, are excluded from the display although they are commonly used in various ECs and bare boards.

The elemental constitution of a PCB also varies greatly among different PCBs.^[^ ^34^ ^]^ Full‐element analysis of WPCBs is not only time‐consuming and laborious but also lacks standardization. This leads to analytical reports that are difficult to compare, and for some elements to be possibly ignored (such as refractory ones, for example).^[^ ^35^ ^]^ Besides, for those CMs used as minor elements, it can be challenging to detect them from WPCBs due to their extremely low concentration (below the ppm). Alternatively, such information may be acquired from electronic manufacturers, but since it is often claimed as a proprietary piece of information, it is not readily accessible. Therefore, a more holistic knowledge of CM constitutional information is obtained by combining the available information from both electronic manufacturing^[^ ^36^ ^]^ and downstream recycling sources,^[^ ^37^ ^]^ as summarized in **Figure** **2**. Since the elemental composition of ECs is more complex than that of bare boards, where CMs are mostly base metals used to make up alloys for conducting propose, the following highlighted CMs are usually found in ECs.

In active components, semiconducting materials are extensively found, and they are composed of Ga, Si, Se, Ge, and other elements in smaller quantities. Amongst, Ga exists in the form of gallium nitride (GaN), gallium arsenide (GaAs), and gallium phosphide (GaP) largely used in power transistors, optoelectronic components, and semiconductors.^[^ ^37d^ ^]^ Indium can be found in the form of InGaN in diodes, transistors, and other semiconductors besides the major use of indium tin oxide in LCD units. Integrated circuits (ICs), known as chips, represent cutting‐edge manufacturing. A single IC is a complex layering of semiconductor wafers, copper, and other materials, which interconnect to form miniaturized transistors, resistors, or other components in a circuit, thus containing a much greater variety of CMs. Intel, IBM, and other semiconductor manufacturers utilize Hf‐based compounds as insulators in the gates of transistors for ICs in the 45 nm (and smaller) generation.^[^ ^38^ ^]^ Most ICs are likely to contain Co which provides ideal wear resistance and electrical resistivity.^[^ ^39^ ^]^ In the case of some PCBs where a real‐time clock needs to be powered, Li‐ion coin‐cell batteries (e.g., CR2032) can be found. Besides, Ni‐metal hydride batteries incorporating REEs are widely used in consumer electronics. However, these rechargeable batteries are supposed to be removed and subjected to separate recycling before the disassembly of PCBs. However, the feedback we got from recyclers, as well as our own experience, indicate that there are always some of these batteries that remain on PCBs, which represent a significant potential hazard, in case of their spontaneous accidental combustion.

Resistors, capacitors, and magnetic (inductive) devices dominate the use of passive components. Common metal film resistors are usually coated with NiCr. Thick film resistors became popular during the 1970s, and most surface‐mounted resistors today are this type. Thick film materials are manufactured using screen and stencil printing processes and vary in the constitution, such as ruthenium oxide (RuO_2_), lead oxide (PbO), bismuth ruthenate (Bi_2_Ru_2_O_7_), or bismuth iridate (Bi_2_Ir_2_O_7_).^[^ ^36b^ ^]^ Nb and Ta are commonly found in electrolytic capacitors. While Ta was the preferred material for these capacitors until 2000, an increase in its price during the early 2000s led to a gradual material transition from Ta to Nb.^[^ ^19b^ ^]^ Li‐ion capacitors exhibit favorable characteristics suitable for applications requiring high energy density, high power density, and exceptional durability. Therefore, Li compounds such as Li_4_Ti_5_O_12_ can be found in the anodes of such capacitors.^[^ ^36c^ ^]^ In the ceramic capacitor sector, the ferroelectric material BaTiO_3_ has garnered considerable attention over the past decade. Rare‐earth ions at low dopant levels, including La, Nd, Sm, Gd, Dy, Ho, Er, Y, etc., are often incorporated into the commercial formulations of BaTiO_3_‐based ceramics to improve the reliability and electrical properties of such components.^[^ ^36d–g^ ^]^ The quantities utilized are substantial, with approximately 10% of the Nd and Dy market, for instance, allocated to the production of ceramic capacitors.^[^ ^36^, ^40^ ^]^ Other ingredients, such as CaZrO_3_, may also be employed in the formulations of certain dielectrics, such as the “X7R” type.^[^ ^36h^ ^]^ Rare earth oxides also play a vital role in functional ceramics, such as microwave dielectric and piezoelectric ceramics. Inductors are usually simple in their structures, consisting of a coil of conducting materials (typically Cu alloys) that either loop around the air or ferromagnetic material.

In addition to those ECs mentioned above, quartz is the most common material for oscillator crystals and is produced with low content of Al, alkali metals (e.g., Li), and other impurities. Cu‐Be alloys are used in electronic connectors where repeated connection and disconnection are desired and such connectors are likely Au‐plated. Be is also likely found in heat sinks as BeO transmits heat very efficiently. Advanced computer processors produce a high heat output which may be removed by W‐Cu heat sinks. For solders, Pb‐free alloys are almost exclusively used today in consumer electronics due to regulatory requirements (e.g., the Restriction of Hazardous Substances Directive in the EU). These solders may contain Sn, Cu, Ag, Bi, In, Zn, Sb, and traces of other metals for commercial use.^[^ ^41^ ^]^ Some CM‐containing materials for more specific electronic applications are not further addressed in detail here.

**Figure 3:**
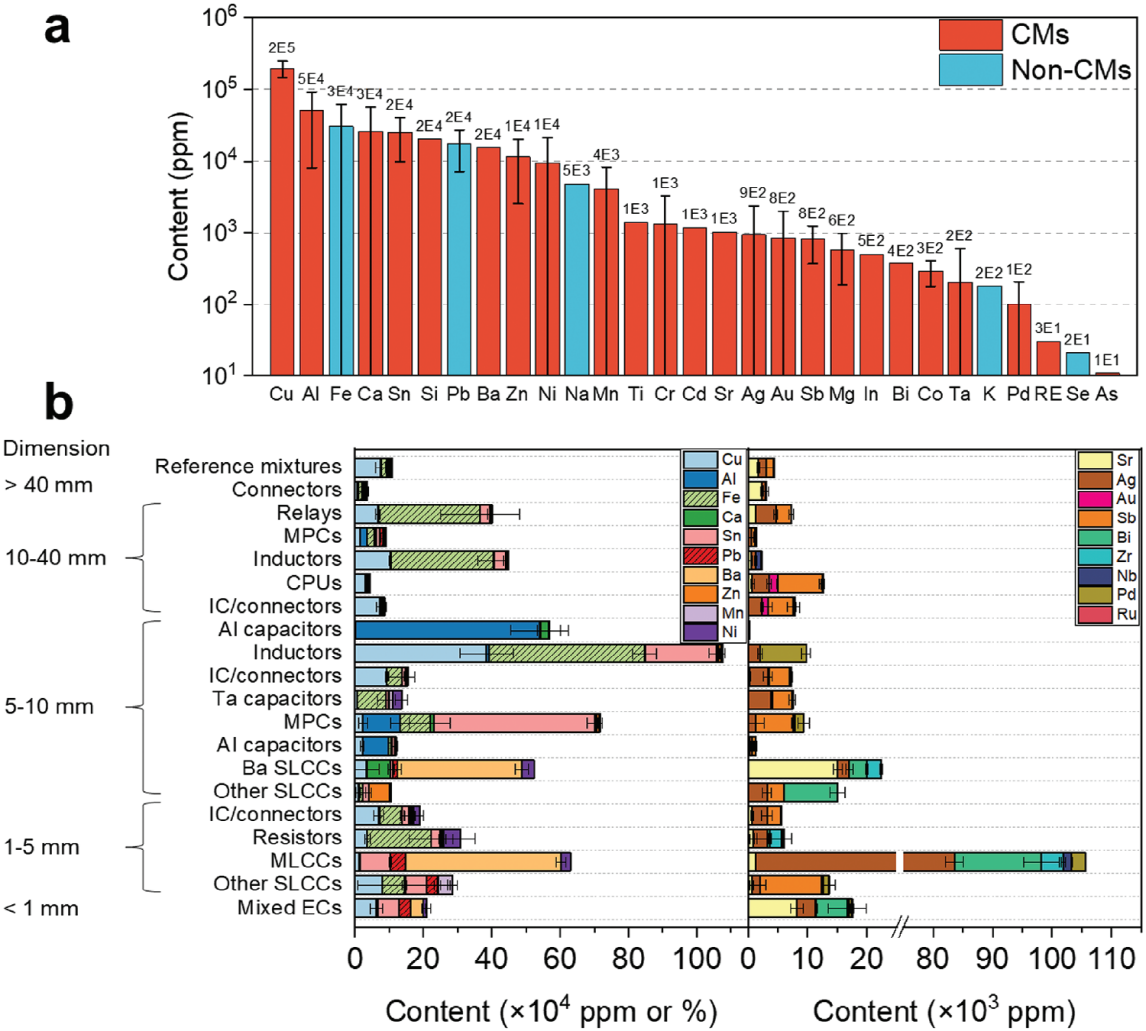
a) Metal contents in WPCBs. Values are averaged from various literatures.^[^ ^37^, ^42^ ^]^ b) Metal contents in individual types of ECs dismantled and sorted from WPCBs. MPCs, SLCC, and MLCCs are abbreviations of metalized polypropylene film capacitors, single‐layer ceramic capacitors, and multi‐layer ceramic capacitors, respectively. Plotted from reference.^[^ ^37f^ ^]^

Overall, a good knowledge of the elemental constitution and metal companionship in different electronic building blocks could lead to a targeted recovery of less content‐rich CMs from PCBs. Some researchers have also provided quantitative analysis data of the metal content, on a PCB level (**Figure** **3**a)^[^ ^37^, ^42^ ^]^ and an EC‐type level (Figure 3b),^[^ ^37f^ ^]^ respectively. For those CMs whose element content ranges between 10 and 10^3^ ppm on a PCB level, their contents may increase by orders of magnitude in a single type of EC. In addition, higher proportions of CMs are prone to present in smaller ECs. Of note, the composition of similar ECs varies with their specificity and manufacturers and may evolve according to the price of raw materials.

### 2.3 Industrial‐Scale Recycling

**Figure 4:**
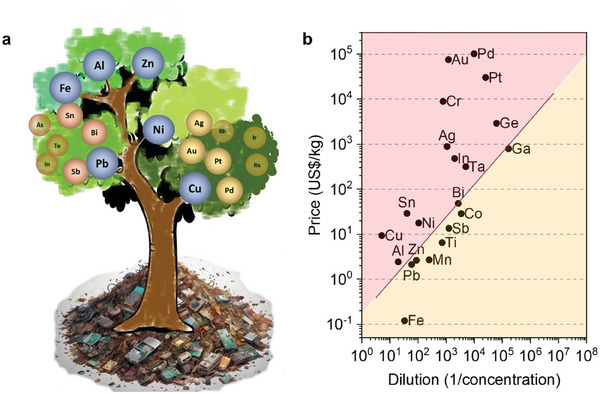
a) A tree diagram illustrates the metal species and their interrelationships recovered from WPCBs through current industrial recycling. The size of the circle where the metal is located approximately reflects how often it is recycled by industries. b) A Sherwood plot shows metals’ concentration in PCBs in relation to their market prices in March 2024.

Although PCBs contain up to 70 elements, the current recycling and refining technology can only recover between six and 17 metals,^[^ ^37^, ^43^ ^]^ including some major metals whose content levels are in weight percentage (wt%) or over grade and some precious metals (e.g., Ag, Au, and PGMs) worth of recovery (**Figure** **4**a). Johnson et al. linked metals’ concentration and recyclability/economics through a Sherwood plot.^[^ ^31^ ^]^ They found that most of the metals that are currently targeted for recycling have concentrations above the metals‐specific Sherwood plot, which is still the case today (Figure 4b), indicating that the “product ore” is typically considered for recovery when a “product ore” grade exceeds the minimum profitable grade of its virgin ore.

For the past three decades, e‐waste recycling industries have embraced pyrometallurgical processes, leading the processes' domination of metal recovery and a major consolidation of e‐waste in a few companies with smelters all over the world till today despite the growing role of hydrometallurgical processes. Steel industries primarily focus on the ferrous fractions of e‐waste for iron recovery, while secondary aluminum industries take charge of aluminum fractions. In smelting operations, WPCBs (populated or unpopulated) are often fed with other metal concentrates or other e‐waste, facilitating the integration of precious metals into a molten copper phase, while the rest are concentrated into slag dominated by lead oxide.^[^ ^44^ ^]^ After smelting, the copper phase goes to a leach‐electrowinning plant for refining copper and precious metals.^[^ ^45^ ^]^ Lead oxide slag, containing Pb, Bi, Sn, Ni, In, Sb, and As, is further treated in a lead blast furnace to produce lead bullion, copper matte, nickel spies, and slag, with each fraction finding its route for refining and applications.^[^ ^18a^ ^]^ Due to similar principles of pyrometallurgy employed, the species of metals that can be recovered industrially are largely similar with minor differences resulting from feed materials and subsequent metal refining processes. The Boliden Rönnskar smelter (Sweden) mainly recovers Cu, Ag, Au, Pd, Ni, and Zn using the patented Kaldo reactor.^[^ ^46^ ^]^ The resulting dust, containing Sn, Sb, In, and Cd, is directed to other operations for further metal recovery. The DOWA group (Japan) enables the recovery of 17 different valuable metals from mixed e‐waste and mineral ores by cutting‐edge smelting and refining technologies and a preeminent metal recycling network. These metals also include Bi, Te, Se, Pt, Rh, Ga, and Ge, besides those metals that Rönnskar can recover.^[^ ^47^ ^]^ Umicore N.V. (Belgium) has established a leading player in metal recovery from various consumer and industrial recyclable products (e.g., electronic scraps, spent catalysts, sweeps, and bullions) and can recover 20 metals.^[^ ^48^ ^]^ China has actively engaged in e‐waste recycling with specialized companies located throughout China to recover various metals, which are still within the regular scope shown in Figure 4a.^[^ ^49^ ^]^

Of all the elements recovered from WPCBs, gold accounts for up to 80% of the value. Therefore, as a valued commodity, gold has been the primary source of interest and driver of recovery. For example, Umicore ensures the recovery of gold from complex e‐waste streams through long‐loop recycling with specialized metallurgical processes.^[^ ^48^ ^]^ Mint Innovation has pioneered to selectively concentrate gold using microorganisms that were identified in abandoned mines.^[^ ^50^ ^]^ The passion for gold recovery has led to the emergence of new technologies, such as oxidative dissolution in organic media, solvent extraction, membrane transfer, supramolecular interaction, biohydrometallurgy, etc.^[^ ^51^ ^]^ Due to economic scale, many recyclers still use the traditional MacArthur–Forrest process (cyanidation) for dissolving gold followed by the Merrill–Crowe process (zinc cementation) for precipitating gold, which causes heavy environmental pollution. Inarguably, electronic manufacturers tend to design products with less and less precious metals over time, instead, they are learning to build gadgets with CMs. This implies an urgency for the development of new recycling technologies, which will otherwise inevitably lead to a massive loss of CMs.

### 2.4 Critical Metals Beyond Current Recycling Capacity

To give a detailed scope of critical metals/metalloids that are spent for being beyond current industrial recycling capacity, we combined the CMs listed in major economies, screened the metals present in PCBs, and subtracted the currently recycled metals, using a set calculation expressed as follow:

CMs = {(US ∪ CN ∪ EU) ∩ (PCB metals) – (Currently recycled metals)} = {Li, Be, B, Mg, Si, Sc, Ti, Cr, Mn, Co, Ga, Ge, As, Sr, Y, Zr, Nb, Ru, In, Te, Ba, Lanthanides (Ln, excluding Pm), Hf, Ta, W, Re, Os, Ir}.

As a result, a total of 41 CMs have been identified. Notably, it could be argued that this list should be taken as a comprehensive complement to current recycling systems rather than one divorced from existing recycling infrastructures. Based on chemical properties, the CMs selected can be classified into alkali and alkaline earth metals, transition and post‐transition metals, and lanthanides. Among them, some CMs are more valued as a wider scale of economies recognizing them, such as PGMs, REEs, and some refractory metals, thus these CMs will be prioritized in this review. The associated recycling/upcycling technologies are presented and discussed in the following sections to tackle the new challenges.

## 3 Metal Enrichment

In PCBs, unlike the major metals illustrated in Figure 3a, most of the CMs are present as minor elements (i.e., concentrations < 0.1%), making their recovery, from the grinder‐based and one‐pot style metal refining processes, a challenging task. On the other hand, these CMs tend to be at much larger concentrations locally in certain electronic materials or applications, forming their so‐called techno‐spheric concentrates (see Section 2.2). Given the complex constitution of WPCBs, simple physical separation or direct size reduction as pretreatment methods are not feasible to access these CMs. To make their recovery economically viable, it is essential to first reduce the waste's composition complexity by its division into enriched fractions, each giving access to CMs above their minimum profitable grades. To do so, the major enabling technologies and constituting strategies for CMs’ enrichment are discussed in this section.

### 3.1 “Look and Pick” Strategy

The first strategy involves the selective disassembly of specific ECs from WPCBs, known as the “look and pick” strategy. First developed for quality inspection for PCB manufacturers, automatic detection of ECs on PCBs quickly gained interest in the recycling industry.^[^ ^52^ ^]^ This strategy is preferably used to identify and disassemble targeted ECs of interest, sometimes to repair PCBs or reuse ECs, rather than dismantle an entire PCB, resulting in a more focused recovery process. Various methodologies have been investigated for EC identification and subsequent disassembly, allowing for optimal efficiency and selectivity.

**Figure 5:**
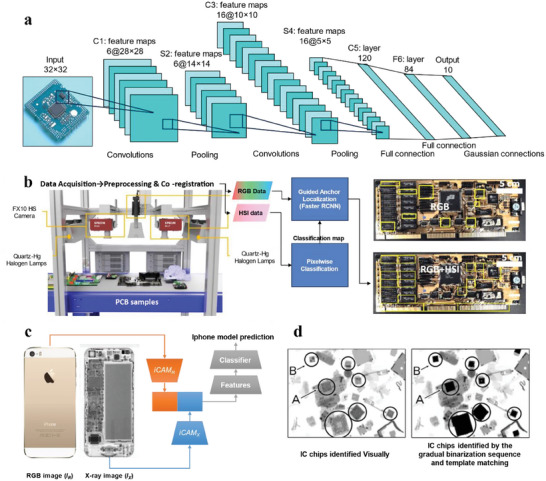
a) A concrete example of LeNet‐5 CNNs. In the architecture, each plane in the convolution layers is a feature map and at each input location, six different types of features are extracted by six units in identical locations in the six feature maps. b) A hyperspectral acquisition setup for ECs’ identification and its workflow of object detection algorithm using a GOL‐based Faster‐RCNN (left). Comparison of the resulting identification performance on the same PCB between the RGB model and the RGB+HSI model (right). Adapted with permission.^[^ ^61^ ^]^ Copyright 2020, IEEE. c) Schematic illustration of the fusion of RGB and X‐ray images for capturing both internal and external features of iPhones and predicting their models. iCAM stands for Iterative Class Activation Mapping network strategy. Adapted with permission.^[^ ^64a^ ^]^ Copyright 2022, IEEE. d) X‐ray transmission images of crushed PCBs in 16‐bit gray scale to show the detection of IC chips by visual and automated means. Reproduced with permission.^[^ ^66^ ^]^ Published under a CC‐BY 4.0 license. Copyright 2020.

The most commonly used method for EC identification is based on optical means, thus simple image segmentation techniques were introduced for this purpose.^[^ ^53^ ^]^ At an early stage, these techniques relied on basic information such as size and location mostly based on edge recognition. With the advent of AI, more precise and relevant optical traits can be obtained from an individual EC. Convolutional neural networks (CNNs) have emerged as a powerful tool for image classification. These machine‐learning algorithms are based on successive layers of neural units, composed of three main types of layers, namely convolutional layers, pooling layers, and fully connected layers (**Figure** **5**a).^[^ ^54^ ^]^ Convolutional layers apply filters, which are small matrices of weights sliding over inputs, to produce feature maps. The weights of the filters are learnable parameters during the training. Then pooling layers are used to reduce the spatial dimension of the feature maps by summarizing the values of a region with a single value, helping to extract higher‐level features. Finally, fully connected layers are used to learn non‐linear combinations of features and map them to desired outputs. Thanks to this architecture, CNNs are well adapted for image classification because convolutions can capture local features in an image such as edges, shapes, and colors. Therefore, the high variability and richness of ECs’ visual traits can be captured and learned by CNNs, making it ideal for the classification of ECs.

As an extension of CNNs, algorithms R‐CNN, where the R stands for the region, are particularly designed for object defection because they enable image detection as well as selection of regions of interest.^[^ ^55^ ^]^ For example, You Only Demanufacture Once (YODO) method, which is based on a faster R‐CNN algorithm and a ResNet50 model, has been applied successfully to retrieve information on laptop models for robotic demanufacturing.^[^ ^56^ ^]^ Some other deep‐learning methods, such as Region Proposal Network (RPN) + Similarity Prediction Network (SPN) + Graph Network (GN), have demonstrated superior performance in recognition to the previously used Faster R‐CNN.^[^ ^57^ ^]^ However, a major challenge in optical identification is the lack of properly labeled data for training recognition algorithms. To address this issue, a self‐training model (YOLOv5) was proposed. It utilizes a teacher model trained on a fully labeled dataset and creates pseudo‐labels based on a second dataset, on which a student model is trained. The student model outperforms the teacher model in detecting eight types of ECs, achieving a precision of 82.3% against 69.6%.^[^ ^58^ ^]^ Nonetheless, relying solely on image information is not always reliable. First, many ECs have similar appearance characteristics, for instance, multilayer ceramic capacitors (MLCCs) versus thick film resistors, chip capacitors versus surface mount diodes, etc. Second, the appearance of ECs can be variable due to dirt, wear, tear, corrosion, and/or damage throughout their product lifetime and post‐consumed recycling process.^[^ ^59^ ^]^ To enhance the reliability of ECs’ identification, labeling recognition was also leveraged, which was initially developed for quality control and counterfeit identification for PCB manufacturers. In this regard, several techniques have been presented for efficient and accurate text detection, such as the Efficient and Accurate Scene Text Detector (EAST) and Character Region Awareness for Text Detection (CRAFT).^[^ ^60^ ^]^

Despite optical identification being recognized as a fast and convenient method, it falls short of providing composition information on ECs. To address this limitation, various approaches have been explored to complement optical identification with additional characterization. For example, a fusion of RGB and near‐infrared data using a guided object localization (GOL) model based on a Faster‐RCNN proves a superior performance over a conventional RGB model in terms of precision and speed (Figure 5b).^[^ ^61^ ^]^ Of note, this is one of the earlier techniques to introduce hyperspectral imaging (HSI), or hyperspectral reflectance imaging to be more specific, for the identification of ECs. Some advanced analytical techniques enable elemental mapping on a bulk PCB at high spatial resolution and fast speed, including laser‐induced breakdown spectroscopy (LIBS), laser ablation‐inductively coupled plasma‐mass spectrometry (LA‐ICP‐MS), and micro X‐ray fluorescence spectroscopy (micro‐XRF).^[^ ^62^ ^]^ LIBS has been integrated in an inverse electronic production line to provide compositional information, such as Cu, W, Ta, Nb, Nd, Au, Pd, and Ag, attributed to their fingerprinted spectra.^[^ ^63^ ^]^ However, these techniques are limited to the surface analysis of samples. Even for LIBS, when it works on an aluminum sample, 10 ablation pulses irradiated at one spot generate a crater with a depth of (1 ± 0.3) mm, whereas for 10 Q‐switch pulses the depth is below 50 µm.^[^ ^63^ ^]^ To tackle this issue, automated X‐ray inspection (AXI) for e‐waste has emerged by virtue of its transmission capacity in recent years. This method has been used to improve the classification accuracy of cellphone's models (Figure 5c),^[^ ^64^ ^]^ defect inspection of solders,^[^ ^65^ ^]^ as well as assess the detachment process of IC chips (Figure 5d).^[^ ^66^ ^]^ Although X‐ray imaging has not yet been used to map PCBs and identify ECs for targeted disassembly, this characterization method shows great potential in providing in‐depth information about ECs.

After identification and targeting the locations of ECs of interest on WPCBs, their disassembly can be processed for either recycling or repurposing. Mechanical methods are the simplest to implement but can be detrimental to the integrity of ECs. Other methods rely on the melting of solder to release targeted ECs at elevated temperatures (e.g., 200 °C heated by hot air or infrared).^[^ ^19^, ^67^ ^]^ To avoid damaging ECs by overheating, an automated laser disassembly process was developed where laser beam can be focused on targeted solder joints to detach ECs without heating the entire component.^[^ ^68^ ^]^ However, this approach does not allow for the retrieval of some surface‐mounted ECs as their solders are not easily accessible. Since each solder point must be targeted individually, this disassembly process can be time‐consuming and can take up to 3 s/EC.^[^ ^35^ ^]^ Additionally, the laser‐based dismantling method requires sophisticated automated devices, thus could only be cost‐effective for high‐value ECs.

In fact, there are not many examples of successful transitions of the “look and pick” strategy.

In the case of Apple, its Daisy and Dava robots can disassemble iPhones to recover valuable materials that traditional recyclers cannot.^[^ ^69^ ^]^ However, these kind of initiatives are still marginal and limited to specific iPhone models and treatment capacities. For large‐scale e‐waste recycling, this strategy shows shortcomings of the slow pace, requirement for expensive equipment, and low universality. Therefore, it is more appropriate to adopt a strategy involving the complete dismantling of ECs from WPCBs, followed by intelligent sorting steps, known as the “dismantle and sort” strategy.

### 3.2 “Dismantle and Sort” Strategy

Maximum liberation and value extraction of ECs from WPCBs can be achieved through the “dismantle and sort” strategy,^[^ ^19^, ^37^ ^]^ also called “evacuate and sort” strategy.^[^ ^70^ ^]^ This strategy involves a single‐step dismantling followed by sorting based on type or chemical composition. The dismantling step can be processed by either mechanical, thermal methods or a combination of the two, as in the “look and pick” strategy.^[^ ^37^, ^71^ ^]^ Besides, chemical desoldering is also feasible through selective acid leaching but involves intensive chemistry.^[^ ^72^ ^]^ Once ECs have been detached from the boards, various methods can be implemented for the sorting into different streams. Through this approach, each stream has a simpler elemental composition and can contain enriched elements of interest, which are much easier to access in downstream recycling steps than within the overall original mixture.

**Figure 6:**
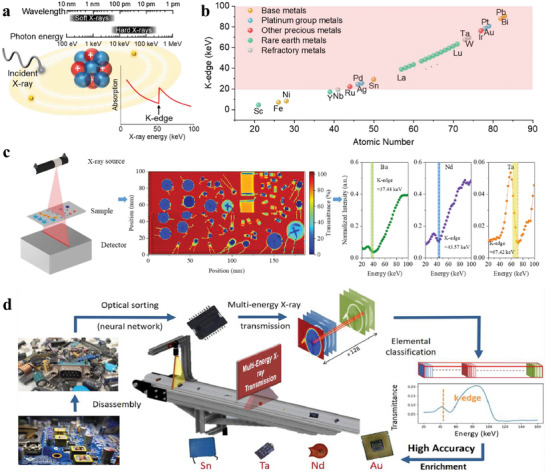
a) Schematic diagram of spectrum region of X‐ray, photoelectric absorption process, and corresponding spectrum with K‐edge marked. b) K‐edge value of selected CMs and other recycled metals as a function of their atomic numbers. Replotted according to the tabular K‐edge data.^[^ ^80^ ^]^ The light red shadow indicates the detectable range of K‐edge set by the state‐of‐the‐art photon counting multi‐energy detector (19.2‐160 keV). c) Schematic illustration of MEXRT detection (left), ME‐XRT mapping image of the detected capacitors (middle), and MEXRT spectra for three representative types of capacitors (right). Adapted with permission.^[^ ^79^ ^]^ Published under a CC‐BY‐NC license, Copyright 2022. d) Schematic flowchart of metal enrichment through the “dismantle and sort” strategy. Reproduced with permission.^[^ ^37f^ ^]^ Copyright 2023, Elsevier.

For the subsequent sorting, optical sorting coupled with AI can yield a high efficiency for sorting ECs.^[^ ^19b^ ^]^ For example, it can reach a sorting accuracy of up to 95% at a conveying speed of 10 m min^−1^ for some designated classifications, yet too slow for an industrial setting.^[^ ^73^ ^]^ Current pure optical means, the same as that used in the “look and pick” strategy, rely on state‐of‐the‐art neural networks for optical image classification to recognize and sort disassembled ECs.^[^ ^74^ ^]^ However, when solely based on optical means, they are unable to realize elemental‐specific classification resulting in limited sorting capability in terms of CMs’ recovery. In addition, the chemical information of materials beneath the external enclosure of ECs is generally of higher interest, which requires characterization techniques should be able to penetrate a certain depth.^[^ ^75^ ^]^ In this regard, X‐ray transmission (XRT) spectroscopy presents as a suitable tool, offering non‐destructive and rapid access to this information. This method has been used in the mining industry to differentiate ores based on the atomic density of targeted elements using single‐ or dual‐energy XRT spectroscopy^[^ ^76^ ^]^ and has been commercialized in the recycling industry.^[^ ^77^ ^]^ The recent development of affordable multi‐energy X‐ray transmission (MEXRT) detectors and their recent commercialization has expanded the capabilities of this tool.^[^ ^78^ ^]^ In X‐ray absorption spectroscopy, the K‐edge is a sudden increase in x‐ray absorption caused by the photoelectric absorption of the photons when their energy is just above the binding energy of the innermost electron shell (K‐shell) of the atoms interacting with them, corresponding to a sharp drop in MEXRT spectroscopy (**Figure** **6**a). Because the concomitant photon energies span the spectroscopic features of most elements, e.g., K‐edge absorption, MEXRT gains more extensive information about the elemental composition (Figure 6b).

Hence, our group developed the first‐of‐its‐kind element‐specific sorting technology taking advantage of MEXRT spectroscopy for more refined sorting after optical sorting, which, for example, demonstrated a 100% sorting accuracy of Nd‐containing ECs, enriching Nd from trace level to 15 wt% (Figure 6c).^[^ ^79^ ^]^ From the acquired energy‐resolved imaging, not only X‐ray transmittance but also MEXRT spectrum can be analyzed to discriminate ECs. Especially, in the optically sorted group mainly composed of SLCCs, it is highly precise to sort into the subgroups by their dominated elements Ba, Nd, and Ta, because of their recognizable K‐edges (37.4, 43.6, and 67.4 keV, respectively). Furthermore, we promoted this technology by integrating optical vision and MEXRT on the same conveyor belt and developing more efficient AI algorithms for this hyperspectral sorting. This system allows to enrich elements up to 10 000 times of its initial content in PCBs and recovers elements that were previously overlooked due to a lack of identification capabilities (Figure 6d).^[^ ^37f^ ^]^

This sorting process not only enhances the concentration of specific elements but also simplifies the composition of the material streams. The reduction in complexity can be evaluated using a Shannon entropy (*S*) equation:

(1) $$S=-k_{B}\sum_{i=1}^{n}x_{i}\log\left(x_{i}\right)$$

where *k*_B_ is the Boltzmann constant and *x*_i_ is the mass ratio of the elements in the mix. It is noteworthy that the entropy of sorted EC stream can be up to ten times lower than that of reference waste PCBs, making it a key to entropy reduction. However, MEXRT sorting still has room to become more powerful by gaining capacities such as discriminating close lanthanides, identifying less concentrated elements, identifying light atomic number elements, analyzing overlapping features, as well as lowering dimensions for data processing. Admittedly, this is inseparable from the simultaneous upgrade of both hardware and software.

### 3.3 Physicochemical Separation

Mechanical‐physical separation is commonly employed as a pretreatment process for enriching valuable metals containing fractions separated from PCBs. This scheme capitalizes on the varying physical characteristics of materials, such as density, magnetic susceptibilities, and electric conductivity.^[^ ^49a^ ^]^ Now a robust system that encompasses WPCB crushing, metals and nonmetals separation, and metals enrichment can boast a high level of automation, cost‐effectiveness, and environmental friendliness. However, these mechanical‐physical methods are only rudimentary to enrich limited metals, such as Fe, Cu, Al, and Ni, and are not specifically designed for the selective enrichment of CMs.^[^ ^81^ ^]^ The pneumatic separation was utilized to segregate components with enriched Ti and Sn content based on different trajectories within vertical airflow. The separation process demonstrated an efficiency exceeding 93% for both categories.^[^ ^82^ ^]^ In the case of a 3‐step separation process combining sieving, magnetic separation, and dense medium separation, several groups of the ECs could be sorted, resulting in metal enrichment of Ti, Mn, Sr, Cr, Nb, Co, Sb, and Bi at enrichment factors of 3.8‐8.8.^[^ ^37b^ ^]^

Some thermal treatments can also facilitate metal enrichment. Capture technology is one of the favorable methods. When recovering waste MLCCs, Ag, Pd, Pb, and Bi are efficiently migrated into an alloy phase (Cu–Ag–Pd–Bi–Pb) by eutectic capture using copper.^[^ ^83^ ^]^ In addition, Ni provided by Ni‐rich MLCCs can be used as an authigenic capture agent, generating a similar metal enrich effect.^[^ ^84^ ^]^ Centrifuge at elevated temperatures was reported to separate solder from PCBs and the CMs contained in solder can therefore be enriched.^[^ ^85^ ^]^ Pyrolysis is also a feasible method to enrich non‐organic fractions from WPCBs by removing organic fractions, as a case of increased concentration of REEs, found in the carbonaceous residue after the pyrolysis of PCBs.^[^ ^86^ ^]^ To suppress the formation of dioxin and furan during thermal treatment, a fast‐cracking method was proposed, enabling the enrichment of some special metals like In, Ga, and Ge, in eutectic melts composed of caustic soda and potassium hydroxide at mild high temperatures (<300 °C).^[^ ^87^ ^]^

**Figure 7:**
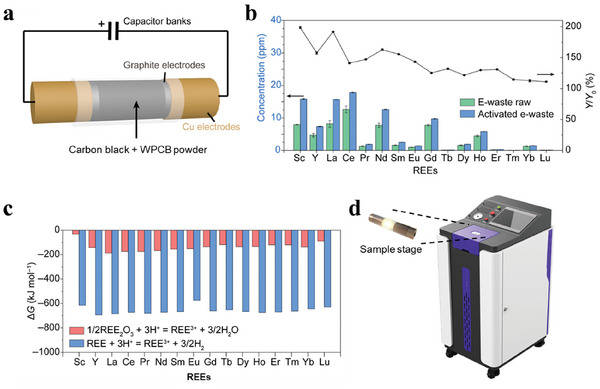
a) Schematic illustration of FJH treatment for WPCB powder. b) Comparison of leachability of REEs in 1 M HCl from raw WPCB powders and the 50‐V FJH‐activated WPCB powders. c) Gibbs free energy change of the rare earth oxides and rare earth metals dissolution reactions in acid. a‐c are reproduced with permission.^[^ ^89^ ^]^ Published under a CC‐BY 4.0 license. Copyright 2022. d) Commercial FJH device with its sample stage (Model JH3.3‐P, Max output current = 500 A, Max output power = 20 kW, Hefei In‐situ Technology. Co. Ltd.).

Recently, an emerging flash Joule heating (FJH) technology has demonstrated some potential in the recycling of CMs with prominent ultrafast, solvent‐free, and sustainable features. This process allows a temperature ramp to ≈3000 °C in milliseconds by ultrafast electrical heating, enabling the evaporative separation of metals from the supporting matrices (carbon, ceramics, and glass).^[^ ^88^ ^]^ Also, this method was used to crack e‐waste matrices (**Figure** **7**a). By species exposure and carbothermic reduction, hard‐to‐dissolve rare earth oxides are converted into highly soluble rare earth metals, leading to the increase of leachability and high recovery yields of REEs (Figure 7b), thanks to the more favorable Gibbs free energy change (Δ*G*) of dissolution reaction in acid (Figure 7c).^[^ ^89^ ^]^ Its robustness versus variable input's composition variability is yet to be studied. In addition, the biggest concern with this technology is up‐scaling. Due to the high current and high‐power requirements, the processing capacity of current commercial equipment is usually <1 g (Figure 7d).

## 4 Metal Separation and Purification

Metal species enriched in solid or liquid formats are usually subjected to separation and purification processes, ending up with the formation of tradeable goods of different purity or grade, such as ingot metals, compounds (oxides, metal salts, etc.), and alloys, so that they can return to their market loop. To facilitate an overview from a forward‐looking perspective of recycling, we discuss different recycling technologies in this section with an emphasis on PGMs, REEs, and refractory metals.

### 4.1 Platinum Group Metals

PGMs are six noble metallic elements (Ru, Rh, Pd, Os, Ir, and Pt) clustered together in the d‐block of the periodic table. Concentrated recycling efforts of PGMs were mainly from spent automotive catalysts as they are a rich source of Rh, Pd, and Pt and account for >50% of total PGM consumption.^[^ ^90^ ^]^ Some metallurgical extraction and solution purification techniques have been well established and bioprocesses have been studied as sustainable alternatives.^[^ ^91^ ^]^ However, much fewer publications are devoted to the recovery of the rare PGMs (Ru, Os, and Ir), especially from WPCBs.^[^ ^51a^ ^]^ Therefore, in this section, we put more focus on these rare, whose potential separation methods could be learned from studies on wastewater,^[^ ^92^ ^]^ synthetic PGM‐containing blends,^[^ ^93^ ^]^ and other waste streams.^[^ ^94^ ^]^

PGMs are commonly chemically inert and refractory due to their unique electronic configurations. Their atoms either have full‐filled states of electrons in the equivalent orbitals, for instance, Os ([Xe]4f^14^5d^6^6s^2^) and Ir ([Xe]4f^14^5d^7^6s^2^), and/or have contraction effect of electrons on the 5s or 6s orbital, for instance, Ru ([Kr]4d^7^5s^1^).^[^ ^95^ ^]^ Therefore, a relatively complete transformation of PGMs into soluble forms requires corrosive media, for example, aqua regia and a mixture of Cl_2_ gas and HCl solution, at the leaching stage.^[^ ^96^ ^]^ However, the formation of trivalent Ir and Ru chloridometalates in >1 m HCl is quite slow in kinetic, affecting their industrial practicability.^[^ ^96a^ ^]^ Alternatively, alkaline fusion is an efficient method to digest Ru powder and anhydrous RuO_2_.^[^ ^97^ ^]^ Peroxide fusion can also convert Ru, Os, and Ir, into solubilized form.^[^ ^98^ ^]^ Since RuO_4_ and OsO_4_ have relatively low melting points and boiling points, they can be readily separated by distillation. Several research teams have dedicated their efforts to developing prospective oxidizing agents for the efficient recovery of Ru and Os from waste materials. These applicable oxidizing agents possess high oxidation potential, including orthoperiodic acid (H_5_IO_6_), potassium periodate (KIO_4_), potassium permanganate (KMnO_4_), sodium bromate (NaBrO_3_), and cerium sulfate (Ce(SO_4_)_2_), among which NaBrO_3_ was found the most efficient at certain conditions, following the chemical equation: 2[RuCl_6_]^3–^ + 2BrO_3_^–^ + 2H_2_O → 2RuO_4_↑ + Br_2_↑ + 12Cl^–^ + 4H^+^ (conversion rate >99% under stoichiometry).^[^ ^99^ ^]^ The resulting RuO_4_ vapor can be recovered in an acid solution, such as HCl. However, the formation of RuO_4_ during the recovery process poses safety concerns due to its explosive nature above 180 °C, which limits its applicability in industrial settings.^[^ ^100^ ^]^ Additionally, ammonium chloride (NH_4_Cl) and thiourea have been adopted to precipitate PGM species in solution and thermal reductive decomposing and electrodeposition have demonstrated validity in producing metallic PGM.^[^ ^101^ ^]^

The extraction methods are of major importance for the hydrometallurgical purification of PGMs as it is in favor of the large‐volume separation of PGM solution rapidly. In practical chloride systems, all PGMs are predominantly present as chloridometalates, which are more commonly recovered through ion‐pair‐type extraction than coordination. Numerous extractants are now available.^[^ ^102^ ^]^ For example, Cyanex 923 is an efficient extractant for the mutual separation of Pt(IV), Pd(II), Ir(IV), and Rh(III) but also for their separation from most of the commonly associated metal ions.^[^ ^103^ ^]^ β‐hydroxyoxime LIX84A is used to separate Pd(II) from Au raffinate in Johnson Matthey and Anglo American Platinum refineries.^[^ ^104^ ^]^ The 4‐alkyl anilines‐HCl solutions system was also used for selective recovery of Rh(III) due to the high stability of Rh/4‐alkyl aniline ion pairs.^[^ ^105^ ^]^ Since [IrCl_6_]^2‐^ is highly extractable while [IrCl_6_]^3‐^ is typically unextractable, the separation of Ir from Pt can be achieved through the redox conversion between Ir(III) and Ir(IV) prior to tri‐n‐butyl phosphate (TBP) extraction.^[^ ^106^ ^]^

The key to the selection of extractants for the isolation of individual PGM requires comprehension of the characteristics of both the substitution of Cl^‐^ with donor atoms of extractants and the ion‐pairing of chloridometalates with cationic extractants. To elucidate the extraction mechanism, some advanced characterization techniques, such as extended X‐ray absorption fine structure (EXAFS) and small‐angle neutron and X‐ray scattering (SANS and SAXS, respective) come in handy to figure out the microscopic structure of a PGM ion in the organic phase.^[^ ^107^ ^]^ Sometimes, however, only through experimental means it is difficult to deduce the extraction mechanism, owing to a lack of knowledge of target speciation, poor solubility, no clues obtained through spectroscopy, or ambiguity in experimental results.^[^ ^108^ ^]^ Computational modeling reveals a powerful tool for structural analysis of the outer‐sphere interactions of chloridometalate in a PGM extraction system. For example, density functional theory (DFT) calculations can provide insight into the extractant's protonation mode and the interactions between protonated extractants and chloridometalates.^[^ ^109^ ^]^ Molecular dynamics simulations allow the understanding of the clustering of multiple complexes or their interactions with solvent molecules.^[^ ^110^ ^]^ Integrating computational modeling with experimental measurements can shed light on certain aspects of outer‐sphere coordination, making it one of the most crucial approaches for accurately understanding the ion‐pair‐type mechanism in the extraction of PGM chloridometalates.^[^ ^96a^ ^]^

From the perspective of engineering practice, it is also paramount to have a well‐designed flowchart with multistage extraction to reach the desired purity of individual PGM in solution.^[^ ^111^ ^]^ However, reagent consumption, a third phase forming, flammability, toxicity, and stability of extractants and metal‐extractant complex in processing media are the major concerns that limit the liquid‐liquid extraction towards a higher cost‐efficiency and sustainability.

In the last two decades, ionic liquids (ILs) have drawn increasing attention as solvents or selective extractants characterized by high chemical stability, tunability, and relatively easy recyclability.^[^ ^112^ ^]^ Extensive investigation on phosphonium‐ and imidazolium‐based ILs has advanced the efficient recovery of PGMs.^[^ ^113^ ^]^ For example, trihexyl(tetradecyl)phosphonium chloride (Cyphos IL101) can readily extract Pd and Pt, while being less efficient for Ru and invalid for Rh.^[^ ^114^ ^]^ The tetrahydropyran‐2*H*‐yl‐protectedthiol moiety‐NTf2, which belongs to imidazolium‐based ILs, can extract Pd(II) from HCl solutions by the coordination of the sulfide to Pd(II).^[^ ^115^ ^]^ In addition to solvent extraction, these ILs have also been applied to support materials for solid‐based separations, such as resins, capsules, membranes, and magnetic nanoparticles.^[^ ^116^ ^]^ Despite the many advantages of ILs possessing, the main drawbacks of using them are the complexity of their synthesis, high cost, and potential toxicity, which have precluded their translation into industrial processes.^[^ ^117^ ^]^

To avoid the issues derived from using ILs, deep eutectic solvents (DES) were proposed as they are deemed more environmentally friendly and less expensive.^[^ ^14a,b^ ^]^ DESs are composed of two or more compounds that can engage in hydrogen bonding with one another. As a result of this bonding, these mixtures exhibit a notable reduction in their melting points, making DES a liquid state at room temperature. A novel technique was presented for the selective separation and recovery of Pt(IV), Pd(II), Rh(III), Ir(IV), and Ru(III) utilizing hydrophobic DESs without chelating agents. The process involves extracting the PGMs into a DES based on tetraoctylammonium bromide and carboxylic acid, followed by re‐extraction using ammonium hydroxide and nitric acid solutions. The extraction efficiency of PGMs from 0.1 m HCl was >99.7% for Pt and Pd, 87.3% for Ru, 45.1% for Ir; 28.5% for Rh.^[^ ^118^ ^]^ Despite numerous academic efforts developing these new solvents for metal extraction, there have yet been no commercial breakthroughs because of the failure to satisfy industrial concerns, for example, high costs, high viscosity, difficulty in recycling solvents, low technique readiness level, to name a few.^[^ ^117^ ^]^

**Figure 8:**
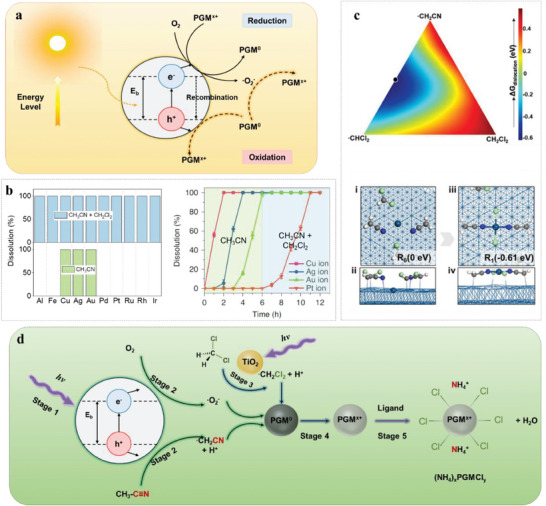
a) Schematic diagram for photocatalytic dissolution of PGMs. b) The dissolution percentages of Al, Fe, Cu, Ag, Au, Pd, Pt, Ru, Rh, and Ir in different solvents under photocatalytic conditions (left) and the dissolution kinetics of Cu, Ag, Au, and Pt from metal catalyst matrix throughout sequential loading of acetonitrile and dichloromethane (right). Reproduced from reference.^[^ ^120^ ^]^ c) Free energy change (Δ*G*_dislocation_) contour plot from the ternary composition diagram for various ⋅CH_2_CN: ⋅CHCl_2_: CH_2_Cl_2_ compositions coordinated to Pt atoms (upper) and the lowest energy structure for R_0_‐Pt and R_1_‐Pt on the Pt (111) surface (lower, Cyan: Pt; Blue: N; White: H; Gray: C; Cyan: Cl; Red: O). Reproduced with permission.^[^ ^121^ ^]^ Copyright 2022, Wiley‐VCH. d) Proposed chemical mechanism for retrieving PGMs by photocatalysis. hʋ, light illumination. Reproduced from reference.^[^ ^120^ ^]^

Notably, the sustainability of the hydrometallurgical recovery will be greatly improved if the process can be realized without the need for strong acids, bases, or toxic organics. The development of the photocatalytic process is undoubtedly an important step in this direction. Previous studies showed the redox potential following the order of Rh (0.75 V_RHE_) < Ir (0.9 V_RHE_) < Pt (1.1 V_RHE_) < Au (1.3 V_RHE_) < TiO_2_ (2.91 V_NHE_), where RHE and NHE stand for normal hydrogen electrode and reversible hydrogen electrode, respectively.^[^ ^119^ ^]^ It indicates that the photogenerated holes are sufficient to oxidize PGM into PGM^n+^, but the chemical inertness and electron‐withdrawing of PGMs impede the occurrence of this photocatalytic oxidation (**Figure** **8**a).

This challenge was tackled by Chen et al.^[^ ^120^ ^]^ They reported such a process utilizing light and photocatalysts such as TiO_2_ for selective retrieval of seven precious metals (Ag, Au, Pd, Pt, Rh, Ru, and Ir) from various waste sources. Remarkably, the dissolution selectivity can be achieved by manipulating solvent and reaction kinetics (Figure 8b). The computed Δ*G*_dislocation_ well elucidates that multiply coordinated R_1_‐Pt states tend to form because of lower energy structures in comparison to singly coordinated R_1_‐Pt states, while the Pt coordinating configuration can be speculated from the lowest energy structure (Figure 8c).^[^ ^121^ ^]^ Over 99% of the targeted elements can be dissolved and the precious metals can be recovered with high purity (≥98%) through a simple reducing reaction. The mechanism involves the excitation of TiO_2_ by ultraviolet light, generating electrons and holes, and stimulating free radical reactions when they react with oxygen and organic compounds, resulting in the oxidation of the precious metal and the formation of solid complexes (Figure 8d).^[^ ^120^ ^]^ The authors also envisaged that water will certainly be more sustainable as a solvent than the current acetonitrile and dichloromethane.

### 4.2 Rare Earth Metals

**Figure 9:**
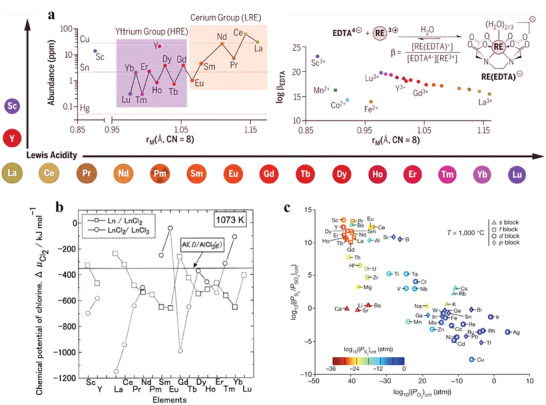
a) Periodic classification of REEs with spheres scaled to their ionic radius and plot of upper crustal abundance of REEs against their ionic radii (left inset). Stability constant of RE(EDTA)‐complexes compared with their ionic radii (right inset). Adapted with permission.^[^ ^1a^ ^]^ Copyright 2019, AAAS. b) Chemical potentials of chlorine corresponding to the equilibria between Ln and LnCl_2_ and LnCl_2_ and LnCl_3_ at 80 °C. Values computed from estimated data with large uncertainty are indicated by dotted symbols. Reproduced with permission.^[^ ^134^ ^]^ Copyright 2000, Springer Nature. c) Oxide‐sulfide anion exchange chemistry exacerbates the thermodynamic differences between metal compounds as illustrated at 1000 °C. Reproduced with permission.^[^ ^135^ ^]^ Copyright 2021, Springer Nature.

Rare earth metals are a group of elements composed of 15 lanthanide elements as well as Sc and Y. The term “rare earth” is bewildering and does not accurately reflect their geological abundance (**Figure** **9**a), but more on their scarcity in terms of economically viable ores and production capacity. Because of the unique and diverse optical, electronic, and magnetic properties they possess individually, the applications of REEs are highly vigorous to motivate modern technologies and devices.^[^ ^1^, ^122^ ^]^ Fearing the lack of a sustainable supply chain and a stable market, many countries or companies are seeking smaller‐scale processes that focus on extracting specific REEs from secondary resources instead of attempting to launch/resume mining operations or large separation units.

As for secondary resources, spent permanent REE magnets can yield the recovery of Nd, Dy, Sm, Pr, and Tb; lamp phosphors offer the opportunity to recover La, Ce, Eu, Gd, Tb, and Y; similarly, nickel‐metal‐hydride batteries for Misch‐metals, including La, Ce, Pr, and Nd.^[^ ^123^ ^]^ All of these recycling initiatives are deemed to save a significant portion of energy and resources concerned from primary mining as well as alleviate the “balance problem” caused by the discrepancy between ore supplies and market needs.^[^ ^123^ ^]^ Although many initial efforts have been made, the recycling rates of REEs from industrial scrap or “urban mines” are still low. In particular, <1% of REEs are recovered from e‐waste (WPCBs) attributed to the scale and techno‐economic concerns.^[^ ^45^ ^]^

Purely from a technical point of view, the predominant challenges of REE separation from REE concentrates are due to the similar chemical characteristics of REEs as a group. Ln^3+^ ions are prevalent in both solution and the solid state in most systems, and the valence 4f orbitals have minor to non‐existent involvement in bonding. In addition, the lanthanide contraction causes a mere 0.15 Å reduction in ionic radii across the series, and the average difference between adjacent elements is merely 0.01 Å.^[^ ^124^ ^]^ Consequently, the combination of predominantly ionic bonding and limited redox activity in aqueous environments leads to their just subtle and gradual variations in thermodynamic properties, explaining the difficulties encountered when employing liquid–liquid extraction (Figure 9a), crystallization, and precipitation methods for intra‐lanthanide separation.^[^ ^125^ ^]^

Nevertheless, such wet processes (hydrometallurgy) involving the use of aqueous solutions and chemical processes to further extract and purify the REEs have gained extensive studies.^[^ ^126^ ^]^ Also, numerous studies are currently underway to explore emerging and cost‐effective methods of recovering REEs from waste streams, including ions exchange and membrane techniques,^[^ ^127^ ^]^ ScCO_2_ extraction,^[^ ^128^ ^]^ ionic liquids extraction,^[^ ^129^ ^]^ biogenic material extraction,^[^ ^130^ ^]^ selective biomineralization with artificial peptides,^[^ ^131^ ^]^ etc.^[^ ^132^ ^]^ As for industrial recycling practices, they are more in favor of proven techniques like scalable liquid–liquid extraction using existing separators. For example, Solvay Group (France) in partnership with Umicore has several lines of REE separation operated a full capacity to treat the secondary resources.^[^ ^133^ ^]^ The main disadvantages of the wet routes are that large amounts of chemicals are required with associated waste effluent to be treated as well as numerous cycles of separation involved.

It is traditionally believed that the direct recovery of REEs from e‐waste is not possible by pyrometallurgical processes because REEs end up as highly diluted oxides in slags in Electric Arc Furnaces (EAFs) or non‐ferrous smelters.^[^ ^11^, ^123^ ^]^ However, the dry process (pyrometallurgy) may provide an easier path with fewer process steps and superposed separation performance in comparison with wet processes for REE‐enriched secondary resources. Uda et al. reported a technique leveraging the distinct disparities in both the redox chemistry of REEs (Figure 9b) and the vapor pressures of their di‐ and trihalides to enable efficient separation not only of REE mixtures but also of mixtures containing REEs and other transition metals (e.g., Co and Fe).^[^ ^134^ ^]^ This technique was demonstrated for permanent magnet recovery with a significantly improved separation factor (*β*_Sm/Nd_ ≈ 570, *β*_Pr/Nd_ = 8.1, *β*_Sm/Dy_ = 2300).^[^ ^134^ ^]^

Another dry method utilizing selective anion exchange was proposed by Stinn and Allanore (2021). They employed various process parameters such as gas partial pressure, gas flow rate, and carbon addition to selectively sulfidize a specific metal from a mixed metal‐oxide feed. By taking advantage of the distinct physical and chemical disparities between sulfide and oxide compounds (e.g., density, magnetic susceptibility, and surface chemistry), vastly enhanced separation can be achieved compared to traditional liquid‐liquid methods.^[^ ^135^ ^]^ The selective sulfidation of metal oxides can be determined by their thermodynamically spontaneous reactions defined by the difference between their relative stabilities given certain conditions, described for oxides and sulfides by a critical ratio of the oxygen and sulfur partial pressures ${\left(P_{S_{2}}/P_{{\mathrm{SO}}_{2}}\right)}_{\mathrm{crit}}$ necessary for the reduction of a given metal compound (Figure 9c). With this principle, they demonstrated various promising applications to f‐block and d‐block element separation required from several mineral processing, among which a combined Ln metals basis purity of 99.7 wt% was achieved in oxide regions from REE magnet recycling.^[^ ^135^ ^]^

Even though numerous studies have demonstrated the feasibility of REE separation and recovery from those well‐known secondary resources using either both wet or dry processes, seldom have they been applied to PCB recycling mainly due to their too high REE dilution and complex composition. A valuable yet overlooked fact is that the use of individual REE is more exclusive in PCBs/ECs, such as the use of Nd in microwave dielectric materials.^[^ ^136^ ^]^ We therefore recently leveraged this benefit and demonstrated the first economically viable Nd recycling process from PCBs.^[^ ^79^ ^]^ These two issues of high Nd dilution in a complex mixture was tackled by taking the “dismantle and sort” strategy (Section 3.2), thus allowing for a fairly simple post‐treatment to be developed, leading to a highly selective method to recover Nd from BaO‐Nd_2_O_3_‐TiO_2_ substrate with easy HNO_3_ leaching and oxalic precipitation. The overall Nd recovery efficiency reaches 91.1% ending up with commercial‐grade Nd_2_O_3_ (>99.6%), and was simulated to prove its economic viability.^[^ ^79^ ^]^ The recycling methods for multiple REEs used as the dopants of MLCCs are more challenging yet of higher value for exploration due to their considerable market share.

### 4.3 Refractory Metals

Refractory metals refer to a set of metallic elements known for their extraordinary resistance to heat (melting points >2000 °C) and wear.^[^ ^137^ ^]^ W, Mo, Nb, Ta, and Re are widely recognized to fit most definitions of the refractory metals. As these elements are members of three distinct groups in the periodic table of elements, they show a wide variety of chemical properties and are found in different electronic materials, thus subjected to various tailored separations.

Among the refractory metals, Ta recycling gains the most attention due to its leveled demand from the electronic industry and profitable price ($150 per kg in 2022).^[^ ^138^ ^]^ Ta recovery from Ta‐based capacitors has been achieved by various combinations of pyrometallurgical and hydrometallurgical processes.^[^ ^139^ ^]^ Some thermal treatments enable the decomposition of organic compounds and Ta enrichment by oxidation/pyrolysis.^[^ ^140^ ^]^ Furthermore, supercritical water (*T* ≥ 374 °C and *P* ≥ 22.1 MPa) was reported efficient and environmentally friendly to remove epoxy resins and recover Ta electrodes ending with Ta purity of 93.2%.^[^ ^141^ ^]^ To further purify the Ta recyclables, additional metallurgical steps are always required. Chloride metallurgy is a classical purification step for many nonferrous metals.^[^ ^142^ ^]^ Using FeCl_2_ as a chlorination agent, Ta can be converted to TaCl_5_, which can be separated by volatility difference and tends to hydrolyze to form TaO_x_.^[^ ^143^ ^]^ Through optimization of the entire recycling route, 93% of Ta can be recycled from Ta capacitor with obtaining tantalum oxide at a purity of over 99%.^[^ ^144^ ^]^ To get a higher grade of Ta oxide, a hydrometallurgical route was reported using pressurized leaching with harsh HF followed by solvent extraction (Alamine 336, pH∼1) and precipitation (NH_4_OH) and it resulted in 99.9% pure Ta_2_O_5_.^[^ ^139a^ ^]^ By contrast, some other acids other than HF may allow selective removal of impurity materials while remaining Ta in its metallic form leveraging the anti‐corrosive nature of Ta to these acids. A two‐stage acidic leaching route (HCl+HNO_3_) can lead to Ta recovery up to 98% with Ta metal purity of 99.9%.^[^ ^145^ ^]^

Nb and Ta have essentially identical ionic radii, thus they exhibit fundamentally similar chemical properties, reactivity, and hardness, making their separation challenging both chemically and physically.^[^ ^146^ ^]^ Refined Nb can also be found in electrolytic capacitors as a substitute for Ta, particularly in years when Ta prices have risen sharply. Thereby some methods analogous to Ta recycling can be expected for recycling Nb from Nb capacitors. However, research efforts into Nb recovery have rarely been reported, probably because of its lower value and the current smaller volume of Nb‐rich waste streams at the end of life.^[^ ^19b^ ^]^

The same reason applies to W recycling. A suitable emulsion liquid membrane process was developed for the separation and recovery of W(VI) from PCB recycling unit wastewater containing low concentrations of W (600 ppm).^[^ ^147^ ^]^ Using Aliquat 336 in hexane as a carrier and sodium hydroxide as a stripping agent, the separation factor for W(VI) versus other co‐ions was found high enough for selective recovery of W(VI). Under optimized conditions, 80% of W extraction was observed after enrichment in stripping phase 4 rounds.^[^ ^147^ ^]^

Rhenium is scarcer and thus more valuable than the other refractory metals. Its excellent physiochemical properties make Re suitable for applications in progressive industries with 80% of Re being used as a component of superalloys.^[^ ^148^ ^]^ The extraction and recycling technologies of Re from secondary resources have been well classified and reviewed, covering the main Re secondary resources including Ni–Re superalloys, W–Re and Mo–Re alloys, and spent Re‐containing catalysts.^[^ ^149^ ^]^ Such recycling normally requires a multi‐step process combining pyrometallurgical and hydrometallurgical techniques.^[^ ^149^ ^]^ Barely any publications were found involving Re recycling from WPCBs although Re alloys are also used for semiconductors, electronic tube components, and electrical contacts.^[^ ^150^ ^]^ It is worth noting that a photochemical approach can be feasible to recover and purify Re from aqueous solutions. In the photochemical reaction system luminated by UV–Vis light with the presence of 2‐propanol and acetone, ReO_4_^‐^ undergoes pre‐complexation with acetone, accelerating electron transfer from 2‐propanol to the excited‐state Re species to form ReO_3_ and ReO_2_ as a precipitate.^[^ ^151^ ^]^ Selective photo‐precipitation of Re was observed by regulating pH and Ru recovery be can achieved up to 90%.^[^ ^152^ ^]^

## 5 Metal Upcycling into Catalysts

**Figure 10:**
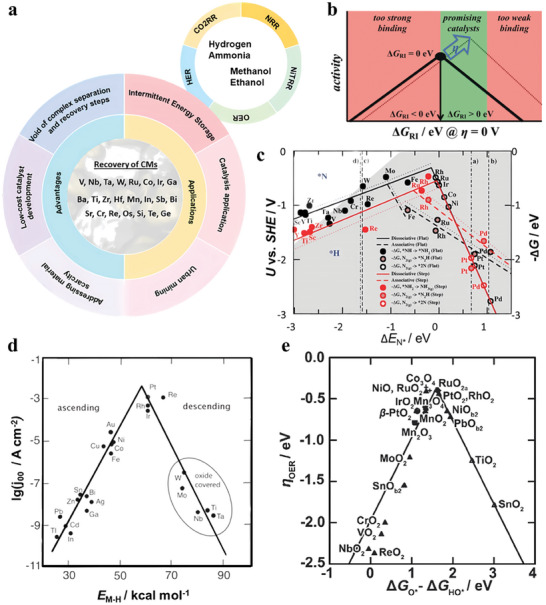
a) Overview of the possible advantages and upcycling application for the recovered CMs from WPCBs. b) Prototypical volcano plot demonstrating the activity of a catalyst plotted against the free binding energy of reaction intermediate (denoted as RI) at 0 overpotential. Reproduced with permission.^[^ ^171^ ^]^ Copyright 2020, John Wiley & Sons, Inc. c) Volcano plots for transition metal catalysts with different surfaces (stepped and flat) in electrochemical NRR applications. Reproduced with permission.^[^ ^172^ ^]^ Copyright 2012, Royal Society of Chemistry. d) Volcano plot by Trasatti for catalysts assessed in HER under acid conditions. Reproduced with permission.^[^ ^173^ ^]^ Published under a CC‐BY 4.0 license. Copyright 2014. e) The volcano plot demonstrating various metal oxides used in OER applications. Reproduced with permission.^[^ ^174^ ^]^ Copyright Elsevier 2022.

While recycling CMs from WPCBs is vital for unlocking untapped secondary resources, the vitality in this field can be more revealed by upcycling. Upcycling, unlike recycling, involves the innovative conversion from waste materials to new products of higher perceived value than the original one.^[^ ^153^ ^]^ Environment and energy are the two critical interlinked problems for promoting social development and CMs play a vital nexus role between these two (**Figure** **10**a). As of 2018, ≈79.5% of the energy economy still relied on conventional energy sources that are not renewable or sustainable.^[^ ^154^ ^]^ With the recent shocks in energy prices, there lies uncertainty in the future of global energy prices, spurring many governments all around the world to seek renewable and cleaner alternatives to back up their energy securities.^[^ ^155^ ^]^ Therefore, an excellent entry point is to directly convert CMs into catalytic materials for the development of clean energy, such as hydrogen, ammonia, methanol, and ethanol. This strategy will not only extend the recycling chains with more economic incentives but also compensate for the material scarcity and high‐cost issues faced by catalyst suppliers, solidifying the CMs’ circularity.^[^ ^156^ ^]^ Such energy storage systems can coordinate well with current energy grids concerning off‐peak issues.^[^ ^157^ ^]^ In this section, we highlight the fundamental considerations for catalyst designs for energy storage and the involved CMs available from WPCBs, as well as some notable works for feasible adaptions of recovered CMs as substitution of reagent CMs for electro‐ and photo‐catalyst synthesis.

### 5.1 Electrocatalyst Design

Electrocatalysis offers notable advantages in terms of precise control, due to the system being closed and cyclic which enables directional electron movement with just an application of external voltage. Here, an efficient catalyst is equally important for the reduction of the activation energy of the catalytic reaction, rendering it more viable.^[^ ^160^ ^]^ However, the performance is highly dependent on the choice of materials as well as catalyst design. Among the various energy storage candidates, hydrogen is highly cited as an efficient energy storage medium owing to its high energy density and the reliable pairing of water electrolysis with renewable energy.^[^ ^161^ ^]^ Fundamental research on water electrolysis, also known as H_2_ evolution reaction (HER), has gained significant traction in the past 10 years. In the early stages of HER research, PGMs and Au were commonly studied in both acidic and alkaline conditions and were deemed to be active and highly stable materials for these reactions.^[^ ^161^, ^162^ ^]^ Subsequently, other catalytic reactions that generate compounds with energy storage capabilities such as ammonia and methanol were also explored.^[^ ^163^ ^]^ Like water electrolysis, noble metal‐based catalysts also exhibit outstanding performances. As such, in most industrial chemical processes, reactions are mainly catalyzed by heterogeneous catalysts that are modeled after noble metals such as Au, Pd, Pt, Ru, Ir, and Rh,^[^ ^162^, ^164^ ^]^ while the raw materials required can mostly be found in WPCBs.

The selection of materials in catalysts is in accordance with the Sabatier principle, which indicates that the bond strength between catalysts and reactants should be balanced to encourage subsequent reactions as depicted in Figure 10b. Over the years, the widespread adoption of density functional theory (DFT) has transformed the calculation of binding energies into a common procedure, thereby elevating the Sabatier principle from an empirical guideline to a quantitative predictive tool. Plotting the reactivity against the activity of catalyst materials yields a distinctive peak‐shaped curve, commonly referred to as the Sabatier plot or volcano curve (Figure 10c–e). This graphical representation effectively highlights the disparity in reactivity and activity among different catalysts. Reactivity, determined by the binding energy of crucial intermediates, signifies the extent of interaction between catalysts and reactants, with positive values indicating weak interaction and vice versa.^[^ ^165^ ^]^ Since catalyst design is the key to efficient catalysis, the Sabatier principle has become a fundamental criterion for design considerations and assessment of material suitability.^[^ ^165a^ ^]^ This principle can also navigate the choice of certain WPCB‐derived products, such as a type of sorted EC, leachate, recovered metals, or their compounds as the raw materials for the catalyst synthesis. In the case of HER, Pt as well as various other precious CMs are observed to be the better candidates for catalysts as shown in the volcano curve.^[^ ^166^ ^]^ However, the scarcity of these metals leads to high costs of employing them as electrocatalysts.^[^ ^167^ ^]^ Therefore, highly efficient, and yet low‐cost catalysts are very desirable.

In many reviews and existing work to find cheaper alternatives, non‐noble metal‐based electrocatalysts are highly sought after as a form of research area due to the possibility of achieving low‐cost catalyst designs.^[^ ^168^ ^]^ Transition metals are often excellent electrocatalyst candidates for their variable oxidation states, which allow them to participate in redox reactions. This ability is crucial in electrocatalysis, where electron transfer is a fundamental process. Transition metals also have d‐orbitals in their electron configuration, which allows them to form various coordination complexes. These d‐orbitals can interact with reactant molecules, facilitating electron transfer and catalyzing reactions.^[^ ^164^, ^169^ ^]^ Despite these advantages, the specific choice of transition metal catalysts depends on targeted reactions, operating conditions, and other factors. Researchers continue to explore and design new transition metal‐based catalysts to improve the efficiency of electrocatalytic processes and the stability of electrocatalysts. Such non‐noble CMs often include Co, Mo, Mn, Cu, Bi, and Zn, where these materials are commonly adapted into various kinds of nanostructured materials and are modified toward the desired catalytic reaction by altering the structure, phase, crystal facet, defects, and morphology.^[^ ^164^, ^169^, ^170^ ^]^ Undoubtedly, this research trend provides ideas and inspiration for the upcycling of CMs that are more abundant in WPCBs.

### 5.2 Photocatalyst Design

Photocatalysis offers the advantages of mild reaction conditions and does not require external energy to operate.^[^ ^159^ ^]^ Like electrocatalysis, a rational catalyst design also plays an important role in the reduction of the activation energy. Moreover, photocatalysis also encounters several challenges, including the lack of control over electron transfer, leading to reduced selectivity in the formation of reduction products, and the vulnerability of semiconductors to photo‐corrosion. When examining catalysts employed in photocatalytic reactions specifically, factors such as charge recombination, the occurrence of back oxidation reactions, and limitations in mass transfer are the primary obstacles impeding the achievement of high efficiency.^[^ ^175^ ^]^ For instance, in photocatalytic HER, the photocatalyst must adhere to specific fundamental requirements (e.g., suitable band positions and energies). Additionally, it should possess the capability to absorb light energy, leading to the generation of electron‐hole pairs that enhance the rate of hydrogen production at a reasonably accelerated pace.^[^ ^161^, ^176^ ^]^ As such, Pt is often used as a main photocatalyst or a co‐catalyst to facilitate HER and as active sites for the back oxidation of H_2_ and O_2_.^[^ ^177^ ^]^ While Pt is considered the most desirable option, many other CMs‐based photocatalysts have also been discovered to show comparable efficiencies. Some examples include CMs such as Sr, Ti, Ga, Zn, Ta, In, and Mn.^[^ ^175^, ^178^ ^]^

Similarly, for photocatalytic CO_2_ reduction reaction (CO2RR), desirable photocatalysts should inherently possess outstanding activity, selectivity, and stability. Additionally, they should also demonstrate substantial surface charge carrier separation to ensure a high degree of electron‐hole recombination, as well as great adsorption ability for the key reactant, CO_2_. In the pursuit of such photocatalysts, a myriad of metal oxide nanomaterials, including ZnO, TiO_2_, In_2_O_3_, Bi_2_MoO_6_, and Co_3_O_4_, have been investigated as promising photo‐responsive materials.^[^ ^178^, ^179^ ^]^ However, these photocatalysts still face many challenges, including sluggish product generation rates, insufficient selectivity, and reliance on sacrificial reagents or additional photosensitizers. In many cases, the addition of co‐catalysts to the system is an attractive method to enhance the photocatalyst. The common co‐catalysts are also noble metal‐based materials such as Pt, Pd, and Ru.^[^ ^175^, ^176^, ^177^, ^179^, ^180^ ^]^ Meanwhile, for photocatalytic ammonia synthesis, comprehensive studies focusing predominantly on TiO_2_ and a range of other semiconductor oxide materials (e.g., WO_3_, ZnO, and Ga_2_O_3_) have revealed their potential as photocatalysts.^[^ ^175b^ ^]^ From the viewpoint of catalyst design, we note the seemingly high importance of upcycling CMs for energy conversion and storage applications.

### 5.3 Waste‐to‐Catalyst Applications

While there is a great wealth of research on the development and synthesis of CM‐based nanomaterials for many catalytic applications, the use of recovered CMs for the same purpose is still in its infancy. With the rising demand for energy storage and green fuels, it is no longer sustainable to simply rely on the primitive methods of manufacturing catalysts. Meanwhile, sheer volumes of CMs present in WPCBs are lost if not properly capitalized, so we acknowledge the significant opportunity should come with this e‐waste. This “urban mining” of e‐waste for valuable metal resources could help us break through the traditional idea of metal separation and utilize the e‐waste to construct catalysts for energy storage applications. Therefore, we wish to highlight some useful synthesis methods to directly utilize the recovered CMs in the abundant e‐waste for low‐cost synthesis of advanced functional catalytic materials.

**Figure 11:**
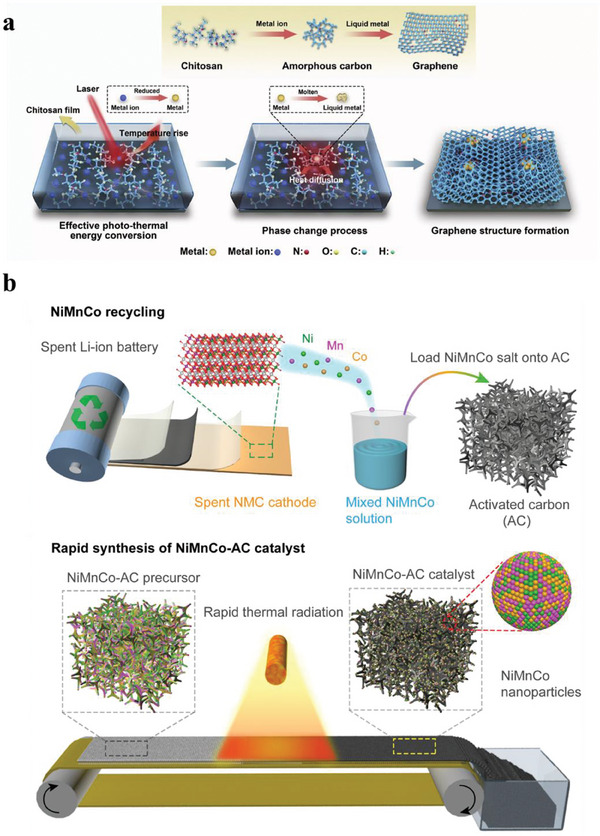
a) Fabrication process for M‐LIG which highlights the formation mechanism in which M‐LIG is formed on chitosan film. Reproduced with permission.^[^ ^181^ ^]^ Published under a license 4.0 (CC BY‐NC‐ND). Copyright 2023. b) Schematic illustration of the recovery process of Ni, Mn, and Co from spent Li‐ion battery cathode and the synthesis process of NiMnCo‐AC catalyst via thermal radiation. Reproduced with permission.^[^ ^156c^ ^]^ Published under a license 4.0 (CC BY‐NC‐ND). Copyright 2022.

In recent years, the synthesis of nano‐catalysts and suitable substrates for catalysis using e‐waste‐derived metal elements was reported in several literatures. One notable example was to convert the recovered CM ions (Zn^2+^, Mn^2+^, Ni^2+^, Ag^+^, Cu^2+^, Cr^6+^, Pd^2+^, and Cd^2+^) into metal‐doped laser‐induced graphene (M‐LIG) using a commercial infrared laser.^[^ ^181^ ^]^ The fabrication process is depicted in **Figure** **11**a and the final M‐LIG product was then used as a catalyst for electrochemical CO2RR in a flow cell. To lower the costs of producing expensive gold‐based catalysts for electrochemical CO2RR into CO product, the recovered gold from waste ECs was utilized as the metal source for catalyst synthesis. Here, Au was recovered by absorbing AuCl_4_^‐^ from the aqueous solution of multi‐metal ions using a 3D cationic framework. The resulting catalyst was further applied in CO2RR experiments under alkaline conditions, which exhibited excellent stability and a high Faradaic efficiency of 95.2% for CO synthesis.^[^ ^156b^ ^]^

A novel technology using iodized tantalum alloy scrap aims to create volatile tantalum(V) iodides. These iodides can then be condensed into fine powders. Through hydrogen reduction in a plasma furnace, these iodide powders can be transformed into high surface area tantalum metal powder precursors. Upon annealing, these precursors yield high‐purity nano powders with consistent particle sizes, minimal oxygen content, and high surface area and capacitance.^[^ ^182^ ^]^ In another work, the in situ preparation of an Nb‐Pb co‐doped and Pd‐loaded TiO_2_ photocatalyst from waste MLCCs was achieved using a facile chlorination‐leaching process. The simulated sunlight photocatalytic H_2_ production rate of the recycled sample could reach 4.4 higher than those of commercial TiO_2_.^[^ ^183^ ^]^ Similarly, by ball milling, the direct conversion of MLCCs into Nb‐Pb co‐doped BaTiO_3_ and Ag‐Pd‐Sn‐Ni loaded BaTiO_3_ nano‐photocatalyst can also be achieved. This resulted in enhanced hydrogen generation (156.7 µmol g^−1^ h^−1^) compared to the commercial BaTiO_3_.^[^ ^184^ ^]^ In addition, magnetic core‐shell Z‐scheme Nb‐Pb‐co‐doped BaTiO_3_/Ni‐Pd@graphite‐like carbon nitride photocatalysts were synthesized from MLCC materials for HER application.^[^ ^156a^ ^]^ The designed photocatalyst displayed a 22.2 times higher HER rate than that of the graphite‐like carbon nitride itself.

To couple the cathodic catalytic reactions, the anodic oxygen evolution reaction (OER) is also investigated using e‐waste‐derived resources. We developed an efficient OER electrocatalyst, NiFe‐Hydroxide, which was derived from the recovered Ni and Fe cations from the waste Class II BaTiO_3_ MLCCs. Here, both leaching and extraction steps were employed simultaneously, followed by ILs extraction to recover Ni, Fe, and Cu cations. The recovered cations mixture was then used to directly synthesize an amorphous anode catalyst, NiFe‐Hydroxide, for hydrogen generation.^[^ ^185^ ^]^ The as‐synthesized catalyst demonstrated much faster OER kinetics than the commercial RuO_2_ catalyst. In another work, as illustrated in Figure 11b, mixed NiMnCo solution can be first obtained by dissolving spent Li‐ion batteries in nitric acid, where the solution can be further loaded on active carbon support and subjected to radiative heating to foam NiMnCo nanoparticles for the anodic OER.^[^ ^156c^ ^]^ Similarly, a simple top‐down leaching strategy was employed to reclaim CMs from cathodes and synthesize single atomic clusters (SACs), which were anchored conductively by the carbon coatings of the cathodes.^[^ ^156d^ ^]^

The successes of these works (summarized in **Table** **1**) have revealed the potential to directly upcycle CMs as catalysts for both photo‐ and electrocatalytic applications as a future solution for energy storage while addressing material scarcity. A key issue is to control its composition by simplifying the input waste complexity. In addition, creating an efficient catalyst necessitates a profound comprehension of three key aspects: the electronic structure of active sites, the morphology of the interface at the molecular scale, and the electrode's interface with ample exposure to the reactants. If these can be achieved, for example, thanks to our “dismantle and sort” strategy, we see limitless opportunities when e‐waste mixture solutions can be refined into catalysts directly, bypassing the complex stages where pure metals must be separated and recovered individually. More than often, such preparations are straightforward and therefore increase the cost‐benefit efficiency of the catalyst synthesis.

**Table 1:** Summary of related works on direct upcycling of metals from waste as catalysts for both photo‐ and electrocatalytic applications.

Recovered CMs	Waste component	Method used	Catalyst	Application Type	Reference
Nb, Pb, Ag, Ba, Ni, Sn, TiO_2_, Pd	Waste ceramic capacitors	Ball‐milling	Core‐shell Z‐scheme Nb‐Pb co‐doped BaTiO_3_/Ni‐Pd‐Ag‐Sn	Photocatalytic HER	[156a]
Ti, Nb, Pb, Pd	MLCCs	Chlorination‐leaching	Nb‐Pb co‐doped TiO_2_	Photocatalytic HER	[183]
Cu (other elements possible, e.g., Mn, Cd, Pb, Zn, Ni)	Wastewater containing heavy metal ions	Laser‐scribing	Cu‐LIG	Electrochemical CO2RR	[181]
Ni, Mn, Co	Lithium‐Ion Batteries	Rapid thermal radiation	NiMnCo‐Activated Carbon	Electrochemical OER/ORR	[156c]
Nb, Pb, Ag, Pd, Sn, Ni, Ba, Ti	MLCCs	Ball‐milling	Nb‐Pb co‐doped and Ag‐Pd‐Sn‐Ni loaded BaTiO_3_	Photocatalytic HER	[184]
Ni, Cu, Fe	Class‐II BaTiO_3_ MLCCs	Green solvent extraction	NiFe‐hydroxide/NiCu‐hydroxide	Electrochemical OER	[185]
Au	Electronic waste with metal ions	Enrichment/Absorption using MOFs	MOF supported Au NPs@1‐_χ_	Electrochemical CO2RR	[156b]
Fe	Lithium‐Ion Batteries	Top‐down leaching	Fe SA/FeO_χ_/FePO_4_	Electrochemical ORR	[156d]

## 6 Conclusions and Perspectives

This review, for the first time, concluded a detailed list of 41 currently unrecycled and therefore spent CMs, which have now the potential to be turned into business opportunities by their recycling from WPCBs, thanks to the recently developed modified recycling paradigm. From a best‐use‐of‐resources perspective, it seems illogical to have millions of tonnes of CMs that have undergone refinement ending up in landfills or being inefficiently recycled because of a disproportionate focus on a dozen prioritized metals among over 70 metals. This list reflects the lag of the recycling industry relative to the development of electronic technology in the context of the global circular economy. From this, a broad array of technologies emerges from the ever‐growing demand for CM recovery following the order of concentration, separation, purification, and upgrading of CMs from the WPCB streams. Among three strategies of metal enrichment, namely “look and pick”, “dismantle and sort”, and physicochemical separation, each strategy has its merits, but “dismantle and sort” demonstrates the most efficacy for CM enrichment at scale, hence the highest potential for profitable recycling process development. Undoubtedly, AI and sensor‐based hyperspectral identification technologies have emerged as crucial enablers to promote element‐specific sorting. Next, the resulting waste streams concentrating on CMs can be further processed with various tailored recycling methods. Here, the recovery of PGMs, REEs, and refractory metals was summarized based on individual chemical and substrate properties. Compared with conventional hydro‐ and pyro‐metallurgical methods, some novel methods, such as photocatalytic dissolution, photochemical precipitation, and selective sulfidation, present higher efficiency, selectivity, sustainability, and potential to scale. Finally, the upcycling of CMs into functional catalysts was presented as a perspective solution to drive sustainable electro‐ and photo‐catalytical synthesis of fuel chemicals. While this upcycling route is still in its infancy, it is inclusive to various CMs and can bypass the complex stages for metal purification, thus gaining higher economic benefits.

The recovery of CMs from WPCBs implies significance not only for e‐waste management and cycle use of finite resources but also for remedying the linked crux between environmental pollution and energy crisis. Therefore, predictable blowout growth of academic efforts will be witnessed following this paradigm‐shifting trajectory in the future, while major impediments shown in the following need to be addressed to promote the transition from proof‐of‐concept research to industrial applications.

1. While numerous initiatives have been suggested to enhance the carbon‐neutrality of PCB design, such as modularized designs and environmentally friendlier substrate alternatives, the inexorable trend indicates that PCBs are continually advancing in electrical performance, integrity, and reliability through material innovation and the ever‐growing intricacy of their nature. Consequently, it is imperative to establish a robust repository of design CMs in PCBs and comprehend the artificial elemental companion relationships thoroughly. Given the extensive range of ECs and PCBs designed for mass production, the analytical capabilities should be bolstered with high‐throughput methods.
2. AI has exhibited desired efficacy in component sorting for both the “look and pick” and “dismantle and sort” strategies, primarily relying on machine vision. When incorporating hyperspectral characterization, such as MEXRT and LIBS, AI can tap into deeper elemental information, enabling element‐specific sorting. Nevertheless, it is imperative to acknowledge that further refinement is required before attaining industrial implementation, including algorithm optimization, detector upgrading, coordination of spatial and spectral information, accurate conveying, multichannel cooperation, cloud database, etc. The overall sorting coverage, accuracy and speed should be iterated to continuously meet the requirements of recycling industries.
3. Multifarious metallurgic methods have been developed for the separation and purification of metals reclaimed from e‐waste. The ultimate goals in general are to obtain metals or their compounds that align well with the grade and impurity requirements for commercial products, while the processes are economically feasible and environmentally friendly. Therefore, chemical similarity and discrepancy of CMs in the periodic system and their specific substrates are the most pertinent for decisive reactions. With the development of quantum mechanics (QM), QM calculations have successfully demonstrated the ability to identify target speciation of metal ions in the aqueous phase and model the organic phase chemistry, which can guide future extractant design and screen candidates for solvent extraction. Since it probes the unique electronic configurations of elements universally, regardless of the f‐block, transition metals, and alkali metals, associated with separation reactions, its role should be highlighted to gain deep understanding and thus precise control on tailored applications. Additionally, the separation process should be designed deliberately in the framework of a circular economy rather than a linear economy.
4. Upcycling of CMs into catalysts is still embryonic and has yet to be sufficiently explored for scalable catalytic applications at lower costs and higher efficiency. Many challenges such as proper choice of CM precursors, use of toxic solvents, high input of resources, and disposal of toxic waste still exist. To emphasize the advantages of material inclusiveness and shorter‐step processing, we also consider some lower selective methods, such as electrodeposition at high potential, in developing useful catalysts from e‐waste, as specific combinations of metals can form alloys of controllable size and structures to be competent for different catalytic applications. In addition, thorough mechanism studies of the catalysts built from CMs should be investigated to provide a complete picture of WPCB utilization in energy conversion application.
5. Proper deployment of those advanced techniques is indispensable for forging an upgraded recycling/upcycling system. The existing recycling infrastructure may lack selectivity or sustainability for recovering CMs, they mostly offer an economical means for processing vast quantities of WPCBs. This paradigm shift in recycling requires the integration of old and new technologies from a whole‐system perspective. Hence, it is necessary to explore mass balance, stoichiometric analysis, thermodynamic and kinetic calculation, energy accounting, cost‐benefit assessment, and waste emissions throughout the system.

## Conflict of Interest

The authors declare no conflict of interest.
